# Heterologous expression of *Spathaspora passalidarum* xylose reductase and xylitol dehydrogenase genes improved xylose fermentation ability of *Aureobasidium pullulans*

**DOI:** 10.1186/s12934-018-0911-1

**Published:** 2018-04-30

**Authors:** Jian Guo, Siyao Huang, Yefu Chen, Xuewu Guo, Dongguang Xiao

**Affiliations:** Key Laboratory of Industrial Fermentation Microbiology, Ministry of Education, Tianjin Industrial Microbiology Key Laboratory, College of Biotechnology, Tianjin University of Science and Technology, Tianjin, 300457 People’s Republic of China

## Abstract

**Background:**

*Aureobasidium pullulans* is a yeast-like fungus that can ferment xylose to generate high-value-added products, such as pullulan, heavy oil, and melanin. The combinatorial expression of two xylose reductase (XR) genes and two xylitol dehydrogenase (XDH) genes from *Spathaspora passalidarum* and the heterologous expression of the *Piromyces* sp. xylose isomerase (XI) gene were induced in *A. pullulans* to increase the consumption capability of *A. pullulans* on xylose.

**Results:**

The overexpression of *XYL1.2* (encoding XR) and *XYL2.2* (encoding XDH) was the most beneficial for xylose utilization, resulting in a 17.76% increase in consumed xylose compared with the parent strain, whereas the introduction of the *Piromyces* sp. XI pathway failed to enhance xylose utilization efficiency. Mutants with superior xylose fermentation performance exhibited increased intracellular reducing equivalents. The fermentation performance of all recombinant strains was not affected when glucose or sucrose was utilized as the carbon source. The strain with overexpression of *XYL1.2* and *XYL2.2* exhibited excellent fermentation performance with mimicked hydrolysate, and pullulan production increased by 97.72% compared with that of the parent strain.

**Conclusions:**

The present work indicates that the P4 mutant (using the XR/XDH pathway) with overexpressed *XYL1.2* and *XYL2.2* exhibited the best xylose fermentation performance. The P4 strain showed the highest intracellular reducing equivalents and XR and XDH activity, with consequently improved pullulan productivity and reduced melanin production. This valuable development in aerobic fermentation by the P4 strain may provide guidance for the biotransformation of xylose to high-value products by *A. pullulans* through genetic approach.

**Electronic supplementary material:**

The online version of this article (10.1186/s12934-018-0911-1) contains supplementary material, which is available to authorized users.

## Background

Aureobasidium pullulans is a promising candidate for generating biotechnological products, such as pullulan, heavy oil, and siderophores [1–3]. Efficient fermentation of xylose is necessary for the biotransformation of lignocellulosic materials to high-value-added products [4]. However, *Aureobasidium pullulans* poorly consumes xylose. The introduction of the xylose reductase (XR) and xylitol dehydrogenase (XDH) pathway or the xylose isomerase (XI) pathway to *Saccharomyces cerevisiae* is a feasible approach to improve xylose fermentation capability [5, 6]. However, whether such strategies can enhance the xylose utilization efficiency of *A. pullulans* remained unclear.

Cofactor preference exists in the enzymes of the XR–XDH pathway [7]. The imbalance of the cofactor between XR and XDH impedes xylose assimilation [8]. The wood-boring beetle-associated yeast *Spathaspora passalidarum* can efficiently utilize xylose for ethanol production [9, 10]. Given that *S. passalidarum* encodes two XR and two XDH with different cofactor preferences, the heterologous coexpression of the most beneficial XR and XDH should be determined. On the contrary, xylulose can be achieved by isomerization of xylose. A large amount of XI genes (XI) has been reported [11], and Maris et al. [12] indicated that XI from *Piromyces* yields high activity. The XI pathway does not exist in *A. pullulans* [13]; thus, the introduction of *Piromyces* sp. XI may accelerate the xylose consumption rate of *A. pullulans*.

**Fig. 1:**
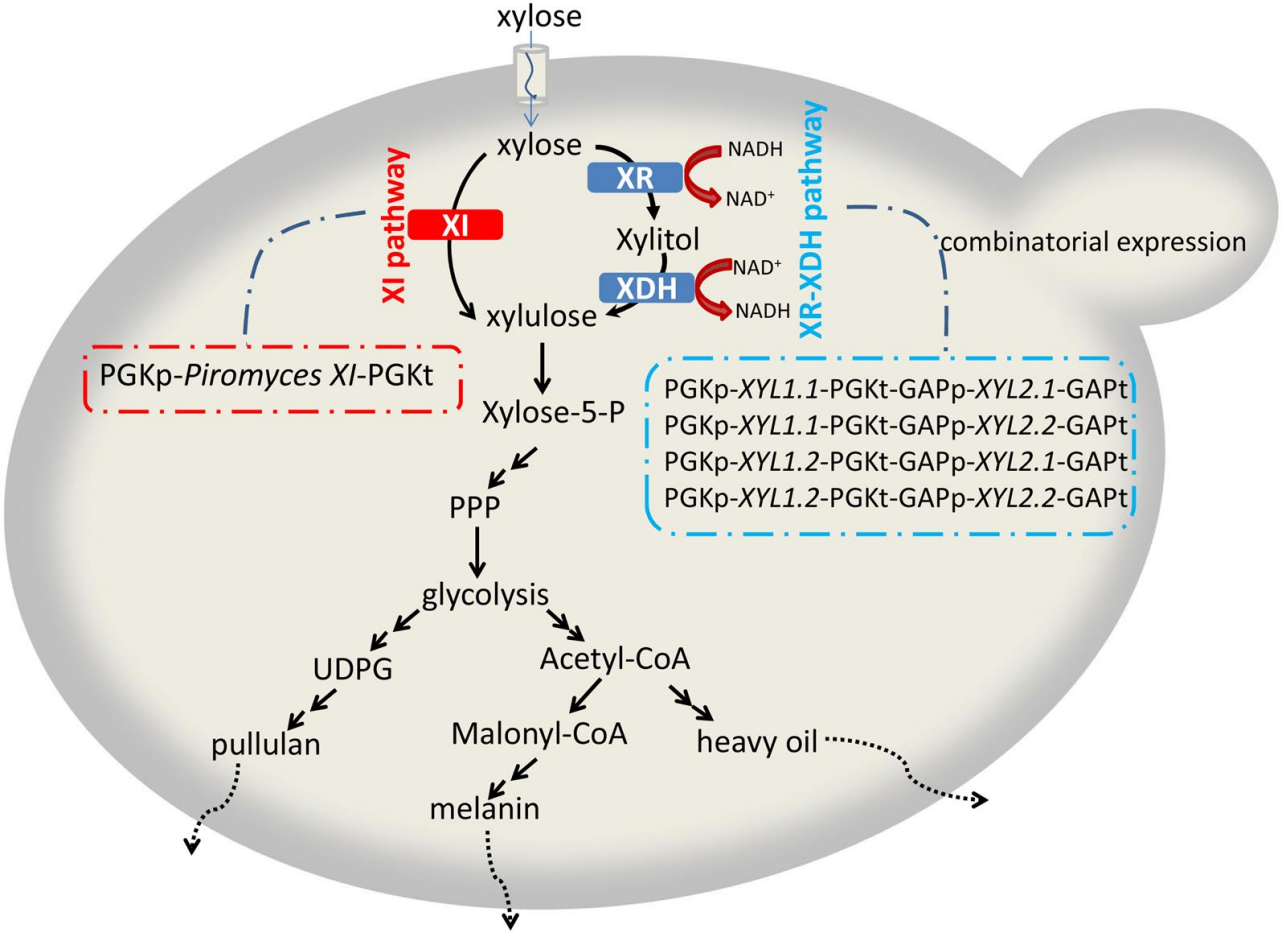
Synthetic biology strategies to enhance xylose utilization in *A. pullulans*. *XI* xylose isomerase, *XR* xylose reductase, *XDH* xylitol dehydrogenase, *PPP* pentose phosphate pathway

Although *A. pullulans* can grow on a variety of carbon sources, its xylose utilization efficiency is poor. Based on our preliminary results, native XR and XDH exhibit low enzyme activity. Hence, the *S. passalidarum* XR–XDH and *Piromyces* sp. XI pathways were constructed (Fig. 1) to improve the xylose-consuming capacity of *A. pullulans*. The xylose fermentation performance of the two different pathways was then compared, and the production of pullulan, heavy oil, and melanin of the mutants was thoroughly investigated. The effects of carbon sources on the fermentation performance of the mutants were evaluated.

## Methods

### Strain and culture conditions

*Aureobasidium pullulans* CBS 110374 was purchased from CBS Fungal Biodiversity Centre. The *A. pullulans* strains were maintained on an YPD agar plate. The YPD agar medium was composed of the following (g/L): yeast extract 10, peptone 20, glucose 20, and agar 20. The inoculum medium contained the following (g/L): carbon source (xylose, glucose, or sucrose) 20, yeast extract 2.0, (NH_4_)_2_SO_4_ 1.0, K_2_HPO_4_ 4.0, MgSO_4_·7H_2_O 0.2, and NaCl 4.0. Initial pH was adjusted to 6.0. The fermentation medium contained the following (g/L): carbon source (xylose, glucose, or sucrose) 50, yeast extract 1.0, (NH_4_)_2_SO_4_ 0.8, K_2_HPO_4_ 6.0, MgSO_4_·7H_2_O 0.4, and NaCl 2.0. Initial pH was adjusted to 6.5. The mimicked hydrolysate contained the following (g/L) [10]: xylose 50, glucose 15, yeast extract 1.0, (NH_4_)_2_SO_4_ 0.8, K_2_HPO_4_ 6.0, MgSO_4_·7H_2_O 0.4, and NaCl 2.0. The pH level was adjusted to 6.5. The fermentation was conducted in 250 mL shake flasks with a working volume of 50 mL at 200 rpm for 168 h.

Seed culture was prepared by inoculating one loop of the respective strain into 250 mL flasks containing 50 mL of inoculum medium and cultivated at 28 °C and 200 rpm for 24 h. A total of 2.5 mL of the seed culture was transferred to inoculate in a 250 mL flask containing 50 mL of fermentation medium and incubated at 28 °C and 200 rpm for 7 days.

### Segment preparation for genetic transformation

In vivo yeast recombination was used for genetically manipulating *A. pullulans* [14]. The primers used are listed in Table S1 (Additional file 1: Table S1). For knock-in of XI gene (GenBank: AJ249909.1), six segments were used to transform *A. pullulans* CBS 110374, and a sketch map was provided (Additional file 2: Figure S1) to enhance the understanding of the transformation process. These six segments comprised the following: rDNAU (upstream of 28S rDNA gene) and rDNAD (downstream of 28S rDNA gene), which were amplified as homologous arms by PCR from *A. pullulans* CBS 110374 28S rDNA gene (Genbank accession number AY139394.1) by using primers 28U1/28U2 and 28D1/28D3, respectively; *HPT* (the gene encoding for hygromycin B phosphotransferase) with primers HPTF and HPTD; PGKp1 (promoter of the gene encoding for phosphoglycerate kinase from *A. pullulans* CBS 110374, JGI protein ID: 59028) with primers PGKpF and PGKpR; XI with primers XI1 and XI2; and PGKt1 (terminator of the gene encoding for phosphoglycerate kinase from *A. pullulans* CBS 110374, JGI protein ID: 59028) with primers PGKtF and PGKt1R.

The knock-in of *XYL1.1* (GenBank: KC818626.1) and *XYL2.1* (NCBI reference sequence: XM_007373204.1) required nine segment as follows: rDNAU (described above); rDNAD with primers 28D2 and 28D3; HPT (described above); PGKp2 with primers PGKpF and PGKp1R; XYL1.1 with primers XR1U and XR1D; PGKt2 with primers PGKt1F and PGKtR; XYL2.1 with primers XDH1U and XDH1D; GAPp1 (the promoter of glyceraldehyde-3-phosphate dehydrogenase gene from *A. pullulans* CBS 110374, JGI protein ID: 82415) with primers GAPps and GAPp1a; and GAPt1 (the terminator of glyceraldehyde-3-phosphate dehydrogenase gene from *A. pullulans* CBS 110374, JGI protein ID: 82415) with primers GAPt1s and GAPta.

The knock-in of *XYL1.1* and *XYL2.2* (NCBI reference sequence: XM_007373986.1) required nine segments as follows: rDNAU (described above); rDNAD (described above); HPT (described above); PGKp2 (described above); XYL1.1 (described above); PGKt2 (described above); XYL2.2 with primers XDH2U and XDH2D; GAPp2 with primers GAPps and GAPp2a; and GAPt2 with primers GAPt2 s and GAPta.

The knock-in of *XYL1.2* (GenBank: KU170767.1) and *XYL2.1* required nine segments as follows: rDNAU (described above); rDNAD (described above); HPT (described above); PGKp3 with primers PGKpF and PGKp2R; XYL1.2 with primers XR2U and XD2D; PGKt3 with primers PGKt2F and PGKtR; XYL2.1 (described above); GAPp1 (described above); and GAPt1 (described above).

The knock-in of *XYL1.2* and *XYL2.2* required nine segments as follows: rDNAU (described above); rDNAD (described above); HPT (described above); PGKp3 (described above); XYL1.2 (described above); PGKt3 (described above); XYL2.2 (described above); GAPp2 (described above); and GAPt2 (described above).

The knock-in of *XYL1.1* required six segments as follows: rDNAU (described above); rDNADs with primers 28D4 and 28D3; HPT (described above); PGKp2 (described above); XYL1.1 (described above); and PGKts1 with primers PGKt1F and PGKt3R.

The knock-in of *XYL1.2* required six segments as follows: rDNAU (described above); rDNADs (described above); HPT (described above); PGKp2 (described above); XYL1.1 (described above); and PGKts2 with primers PGKt2F and PGKt3R.

The knock-in of *XYL2.1* required six segments as follows: rDNAU (described above); rDNAD (described above); HPTs with primers HPTF and HPT1R; XYL2.1 (described above); GAPpS with primers GAPp3S and GAPp1a; and GAPt1 (described above).

The knock-in of *XYL2.2* required six segments as follows: rDNAU (described above); rDNAD (described above); HPTs (described above); XYL2.1 (described above); GAPpS with primers GAPp3S and GAPp2a; and GAPt1 (described above).

### Transformation of *A. pullulans*

Electroporation transformation was used to transform *A. pullulans*. The strain was cultured in YPD medium at 28 °C with 200 rpm for 16–24 h. Pellets obtained were washed by sterile water twice and then washed twice with buffer 1 (1 M sorbitol, 50 mM sodium citrate, pH 5.8). Washed cells were resuspended in 0.5 mL buffer 1, and respective DNA segments were added. The mixture was subjected to electroporation transformation with condition of resistance 200 Ω, capacitance 25 µF, and voltage 1.5 kV. Cell suspension was then incubated at 28 °C with 100 rpm for 1 h and subsequently spread onto the YPD agar plate with 100 µg/mL hygromycin B. Transformants appeared after cultivation for 2–3 days. Positive colonies were purified on the YPD agar plate with 100 µg/mL hygromycin B for further study. The constructed strains were verified by PCR amplification (Additional file 2: Figures S2–S4).

### Enzyme activity measurements

Cells were grown in the fermentation medium with xylose as carbon source and harvested in the exponential growth phase. Cells were washed twice with sterile water, and proteins were extracted using yeast protein extraction reagent (Takara, Beijing, China) according to the manufacturer’s instructions. Protein concentrations were measured using Bradford protein assay kit (Takara, Beijing, China). XI and XDH activities were measured according to the method described by Dmytruk et al. [15]. One unit of XI activity is defined as the amount of enzyme that produces 1 μmol of xylulose per min. One unit of XDH activity is defined as micromoles of NADH (nicotinamide adenine dinucleotide plus hydrogen) reduced or oxidized produced by 1 mg of protein per min. XR activity was measured according to the method described by Bengtsson et al. [16]. One unit of XD activity is defined as micromoles of NADH reduced or oxidized produced by 1 mg of protein per min.

### Analytical methods

Dry cell weight (DCW) and pullulan production was measured according to the method described by Chen et al. [17]. For removing biomass, 6 mL of culture broth was centrifuged. Sediments were dried overnight at 110 °C and determined as biomass. Then, 5 mL of supernatant was mixed with 10 mL of cold ethanol to precipitate the exopolysaccharide. Dry pullulan weight was calculated by drying the precipitate at 80 °C to a constant weight. Melanin production was measured by using the method described by Zheng et al. [18]. After cell lysis by homogenization with 20 mM sodium acetate buffer (pH 4.5) at 5000 rpm for 2 min and centrifugation at 5000×*g* for 10 min to remove cell debris, sediments were dissolved in twofold volume of 0.5 M NaOH at 121 °C. Melanin was precipitated using 0.5 M HCl and dried to a constant weight. The amounts of NAD^+^ (nicotinamide adenine dinucleotide) and NADH were measured according to the method described by Guo et al. [19]. Cells were collected after centrifugation and then resuspended in 300 μL of 0.2 M HCl (for NAD^+^) or 0.2 M NaOH (for NADH). The suspensions were boiled for 7 min, cooled rapidly in an ice bath, and added with 300 μL of 0.1 M NaOH (for NAD^+^) or 0.1 M HCl (for NADH). The mixtures were centrifuged, and the supernatants were used to determine the amounts of NAD^+^ and NADH. Heavy oil production was measured by the method of Liu et al. [2]. Sediments of the culture were mixed thoroughly with 10 mL of a 1:1 methanol–chloroform solution. These sediments were obtained from 50 mL of culture broth by centrifugation at 12,000×*g* and 4 °C for 5 min. The mixtures were subsequently centrifuged under the same condition for 10 min, and then the solvent was removed using a vacuum rotary evaporator at 80 °C. Oil production was calculated after drying. Concentrations of xylose, xylitol, glucose, and sucrose were determined using high-performance liquid chromatography (Agilent 1200) equipped with Aminex HPX-87H, 300 × 7.8 mm column (Bio-Rad, Hercules, CA, USA) and eluted with 5 mM H_2_SO_4_ at a flow rate of 0.5 mL/min.

## Results and discussion

### Strain construction

*Aureobasidium pullulans* can produce several biotechnologically important products, including pullulan, heavy oil, melanin, and a large spectrum of extracellular enzymes [13]. Few native microorganisms are capable of simultaneously consuming xylose and glucose [20]. *A. pullulans* can grow on various carbon sources and simultaneously consume glucose and xylose [17], but its xylose-consuming capacity is low [21]. Thus, *A. pullulans* CBS 110374 was selected for comparing the efficiency of xylose fermentation via *S. passalidarum* XR–XDH and the *Piromyces* sp. XI pathways.

Two heterologous pathways, namely, *S. passalidarum* XR–XDH and *Piromyces* sp. XI, were constructed and compared in *A. pullulans*. The *Piromyces* sp. XI pathway was built in *A. pullulans* by overexpression in XI encoded by *Piromyces* sp. XI gene. The strain of *S. passalidarum* exhibits two XR and two XDH. The *S. passalidarum* XR–XDH pathway was assembled in *A. pullulans* through in vivo yeast recombination [14]. With the combinatorial expression of the two XR and two XDH genes, four recombinant *A. pullulans* strains harboring engineered gene assembly for enhanced xylose metabolism were synthesized. The four strains were P1 (including XR encoded by *XYL1.1* gene and XDH encoded by *XYL2.1* gene), P2 (including XR encoded by *XYL1.1* gene and XDH encoded by *XYL2.2* gene), P3 (including XR encoded by *XYL1.2* gene and XDH encoded by *XYL2.1* gene), and P4 (including XR encoded by *XYL1.2* gene and XDH encoded by *XYL2.2* gene). For comparison, another four strains, namely, a single overexpression of *XYL1.1* (S1), *XYL1.2* (S2), *XYL 2.1* (S3), and *XYL 2.2* (S4) were constructed. The constructed strains are summarized in Table 1.

**Table 1:** Strains used in this study

*A. pullulans*	Relevant genotype	Xylose pathway
X5	rDNA*::PGKp*-*XI*-*PGKt*	XI
S1	rDNA*::PGKp*-*XYL1.1*-*PGKt*	–
S2	rDNA*::PGKp*-*XYL1.2*-*PGKt*	–
S3	rDNA*::PGKp*-*XYL2.1*-*PGKt*	–
S4	rDNA*::PGKp*-*XYL2.2*-*PGKt*	–
P1	rDNA*::PGKp*-*XYL1.1*-*PGKt*-*GAPp*- *XYL2.1*-*GAPt*	XR–XDH
P2	rDNA*::PGKp*-*XYL1.1*-*PGKt*-*GAPp*- *XYL2.2*-*GAPt*	XR–XDH
P3	rDNA*::PGKp*-*XYL1.2*-*PGKt*-*GAPp*- *XYL2.1*-*GAPt*	XR–XDH
P4	rDNA*::PGKp*-*XYL1.2*-*PGKt*-*GAPp*- *XYL2.2*-*GAPt*	XR–XDH

–, single gene overexpression

### Evaluation of xylose utilization and cell growth of the recombinant strains

**Fig. 2:**
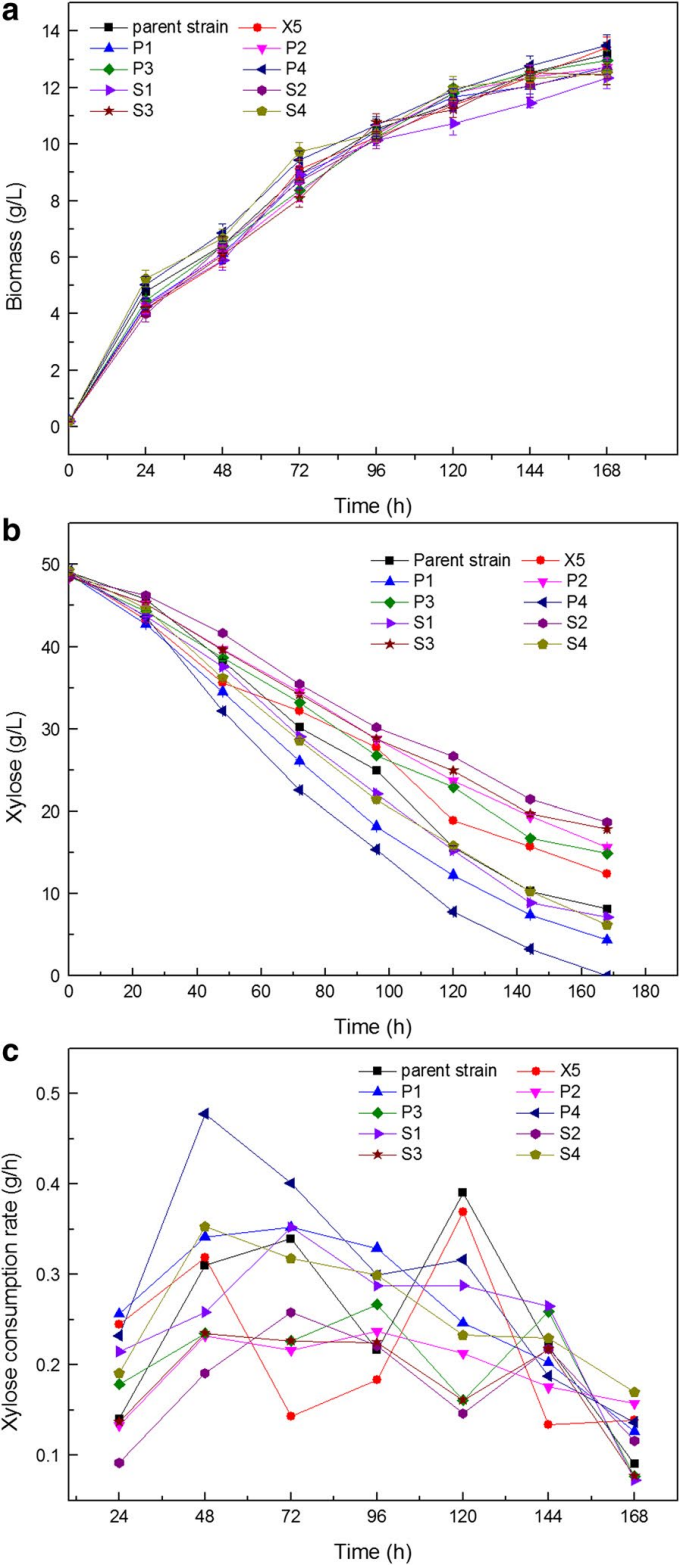
Comparison of cell growth (**a**), xylose utilization (**b**), and xylose consumption rate (**c**) of the recombinant *A. pullulans* with that of the parent strain. Data are given as mean ± standard error value of each group (*p* < 0.05)

The recombinant *A. pullulans* and the parent strain were cultured under the same conditions to compare their xylose fermentation performance. The biomass accumulation of all the mutants showed no obvious difference compared with the parent strain (Fig. 2a). Overexpression of *Piromyces* sp. XI gene in *A. pullulans* CBS 110374 resulted in XI activity of 0.12 U/mg protein (Table 2), but the xylose utilization efficiency was not improved accordingly (Fig. 2b, c). Kuyper et al. [22] also revealed that the sole expression of XI gene in *S. cerevisiae* cannot result in a considerable improvement in xylose utilization. Adaptation or excessive genetic approach may help to overcome this drawback [23, 24].

**Table 2:** Enzyme activity of the recombinant *A. pulluans*

Strain	U/mg protein		
	XI	XR	XDH
CBS 110374	ND	0.08 ± 0.02	0.11 ± 0.04
X5	0.12 ± 0.02	0.05 ± 0.01	0.07 ± 0.03
S1	ND	0.15 ± 0.02	0.07 ± 0.02
S2	ND	0.14 ± 0.01	0.06 ± 0.03
S3	ND	0.07 ± 0.03	0.16 ± 0.03
S4	ND	0.11 ± 0.04	0.78 ± 0.02
P1	ND	0.16 ± 0.03	0.43 ± 0.02
P2	ND	0.13 ± 0.02	0.17 ± 0.01
P3	ND	0.11 ± 0.03	0.15 ± 0.02
P4	ND	0.23 ± 0.02	0.82 ± 0.03

*ND* not detectable. Data are given as mean ± standard error value of each group (*p* < 0.05)

The xylose consumption capabilities of the mutants with single or combinatorial expression of the two XR genes and two XDH genes were examined. As shown in Table 2, XR activities of S1, S2, P1, P2, P3, and P4 increased 1.88-, 1.75-, 2.0-, 1.63-, 1.38-, and 2.88-fold, respectively, compared with the parent strain. The XDH activities of S3, S4, P1, P2, P3, and P4 increased 1.45-, 7.09-, 3.91-, 1.55-, 1.36-, and 7.45-fold, respectively, compared with the parent strain. Consistent with the enzyme activity, the strain of P4 showed the best xylose consumption capacity, whereas P2 and P3 showed the lowest fermentation capabilities. Furthermore, xylose fermentation performance of the strains with single overexpression of these genes was comparable with the performance of strains with double-overexpressed XR and XDH genes (Fig. 2). The highest xylose consumption rate (0.48 g/h) was achieved by P4 (Fig. 2c). The variation in xylose fermentation performances of the strains with combinatorial expression of XR and XDH genes may be attributed to different cofactor preferences [25].

### Internal redox state of recombinant strains with the introduction of the XR–XDH pathway

**Fig. 3:**
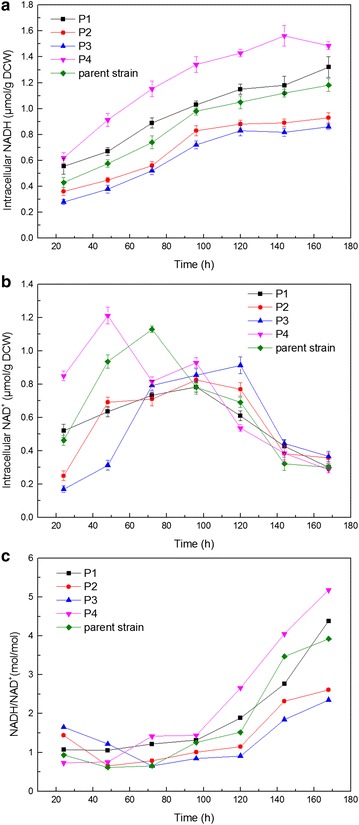
Time courses profile of intracellular NADH (**a**), intracellular NAD^+^ (**b**) and NADH/NAD^+^ (**c**) in *A. pullulans* mutants and the parent strain. Data are given as mean ± standard error value of each group (*p* < 0.05)

The internal redox state of the recombinant strains was investigated from 24 to 168 h during batch fermentation. As shown in Fig. 3, the total dinucleotide pool and NADH/NAD^+^ ratio of P2 and P3 were relatively high during the early period of fermentation, gradually decreasing during the exponential phase. The total dinucleotide pool and NADH/NAD^+^ ratio in the P1 and P4 strains was low at 24 to 48 h and then increased significantly in the latter part of fermentation. Compared with the parent strain, P1 and P4 strains showed decreased NAD^+^ levels and increased NADH levels during fermentation. Thus, the NADH/NAD^+^ ratio of P1 and P4 were relatively high from 72 h. In addition, the strains of P1 and P4 showed superior xylose utilization performance compared with other strains. The P4 mutant, with overexpressed *XYL1.2* and *XYL2.2,* exhibited the best xylose-consuming capacity and the highest intracellular reducing equivalents.

During anaerobic xylose fermentation by *S. passalidarum*, cofactor balance in its XR–XDH pathway is important for xylose utilization (Hou 2012) [26]. The combinatorial expression of XR and XDH genes from *S. passalidarum* in *A. pullulans* CBS 110374 altered the internal redox state of the recombinant strains. Cadete et al. [27] indicated that two XR and two XDH genes from *S. passalidarum* exhibit different affinities for NADH or NAD^+^, respectively; hence, the combinatorial expression of the XR and XDH genes can alter the internal redox state. The obtained results suggested that the strain with increased NADH/NAD^+^ ratio and intracellular reducing equivalents exhibited improved xylose utilization performance.

### Production of pullulan, melanin, and heavy oil by mutant strains with the introduction of XI or XR–XDH pathway

**Fig. 4:**
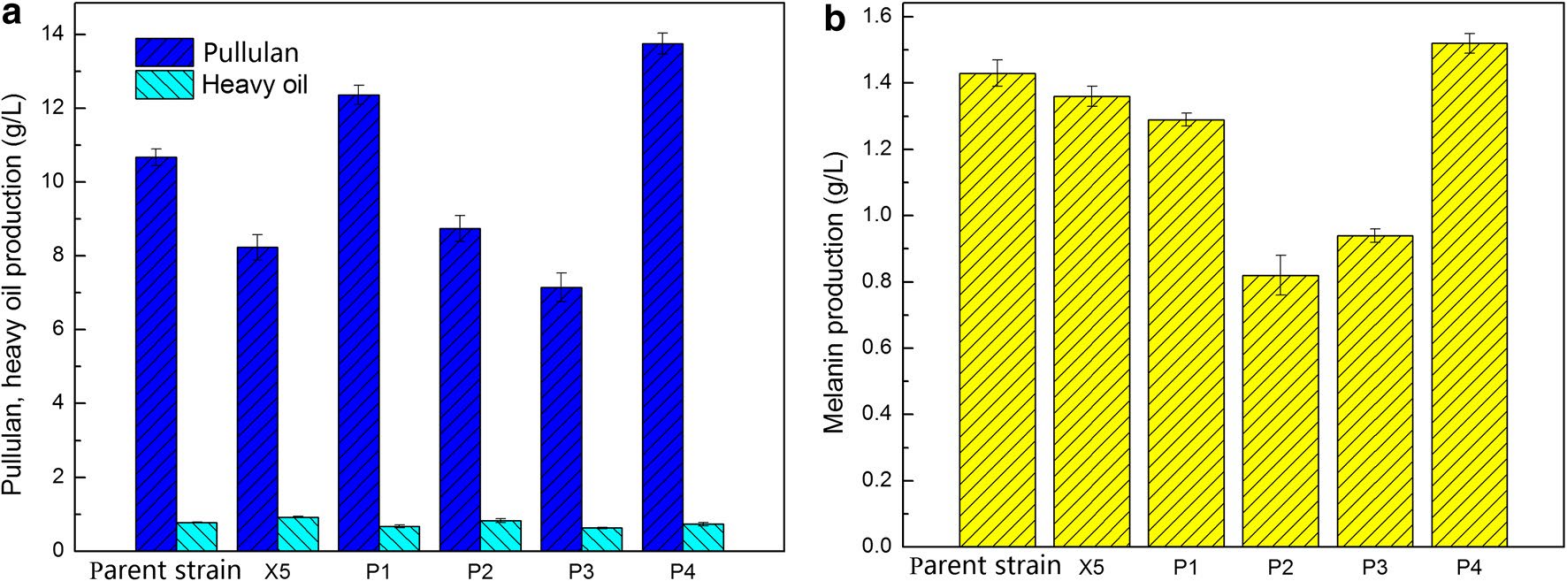
Production of pullulan, heavy oil (**a**), and melanin (**b**) by the mutant strains and the parent strain. Data are given as mean ± standard error value of each group (*p* < 0.05)

The major metabolites in mutants and parent strains were investigated. Compared with the parent strain, P1 and P4 strains showed increased pullulan production to 15.84 and 28.87%, respectively, whereas the pullulan production of X5, P2, and P3 were 22.87, 18.00, and 32.71% lower than that of the parent strain, respectively (Fig. 4a). In addition, the production of pullulan, heavy oil, and melanin of the single overexpressed gene was not comparable with that of the parent strain (Additional file 2: Figure S5). Heavy oil production of all recombinant strains showed no evident difference with the parent strain (Fig. 4a). Melanin production of P4 was 6.29% higher than that of the parent strain, whereas X5, P1, P2, and P3 respectively showed 4.90, 9.80, 42.66, and 34.27% decrease in melanin production compared with that of the parent strain. Figure 2a shows that the biomass of the recombinant strains did not differ from that of the parent strain (may be attributed to limited nitrogen sources), but their pullulan production were markedly varied (Fig. 4a). This result suggested that the difference in xylose utilization can regulate pullulan synthesis.

Despite showing slightly higher XR and XDH activity than the parent strain (Table 2), P2 and P3 produced fewer products possibly because of the low NADH/NAD^+^ pool [19]. The P4 strain showed the highest increase in product formation (especially pullulan production), and the NADH of P4 was excessive after 96 h of fermentation (Fig. 3c). This observation is consistent with those of Su et al. [28], who indicated that polyol accumulation is an alternative approach to regenerate cofactors when NAD(P)H is in excess. Han et al. [29] reported that product formation can be enhanced with increased intracellular reducing equivalents in *S. oneidensis* strains. Hence, the highest intracellular reducing equivalents in P4 may also be beneficial for pullulan production.

Cofactor balance in the XR–XDH pathway is essential for xylose utilization [26], and the two XR and two XDH genes from *S. passalidarum* exhibit different affinities for NADH or NAD^+^ respectively [27]. The heterologously expressed genes (combinatorial expression of *XYL1.2* and *XYL2.2*) consolidated the xylose metabolic pathway (Fig. 2), and a large amount of reducing equivalents can be synthesized through pentose phosphate pathway, thus leading to improved fermentation performance.

### Effects of carbon source on mutant strains with the introduction of the XI or XR–XDH pathway

Three carbon sources, namely, xylose, glucose, and sucrose, were selected for testing the metabolic effects of mutants (Table 3). The pullulan production of all mutant strains utilizing glucose or sucrose showed no marked difference with that of the parent strain. The pullulan yield of the tested strains from sucrose was the highest compared with those of other carbon sources, indicating that sucrose was the most beneficial source for pullulan synthesis. Long et al. [30] also suggested that sucrose is the most appropriate source for pullulan production. However, the highest pullulan production was acquired by P4 strain from xylose, thereby demonstrating that the construction of the *S. passalidarum* XR–XDH pathway in *A. pullulans* can substantially enhance pullulan synthesis. The improvement in pullulan synthesis may be mainly attributed to the increased xylose consumption capacity.

**Table 3:** Comparison the fermentation abilities of the parent strain and the recombinant strains using several carbon sources

Strain	Carbon source	Consumed carbon source (g/L)	Maximum carbon source consumption rate (g/h)	Pullulan (g/L)	Heavy oil (g/L)	Melanin (g/L)	Biomass (g/L)	Pullulan yield (g/g)
Parent strain	Xylose	41.38 ± 0.34	0.39	10.67 ± 0.30	0.78 ± 0.05	1.43 ± 0.04	13.18 ± 0.47	0.26
	Glucose	46.19 ± 0.45	0.61	12.55 ± 0.23	0.94 ± 0.05	0.93 ± 0.03	12.54 ± 0.36	0.27
	Sucrose	48.34 ± 0.36	0.58	13.32 ± 0.18	1.05 ± 0.06	0.87 ± 0.03	13.83 ± 0.42	0.28
X5	Xylose	35.72 ± 0.49	0.37	8.23 ± 0.25	0.92 ± 0.03	1.36 ± 0.09	13.41 ± 0.32	0.23
	Glucose	47.15 ± 0.37	0.62	11.53 ± 0.34	0.85 ± 0.04	0.76 ± 0.03	13.86 ± 0.35	0.24
	Sucrose	46.89 ± 0.38	0.59	13.25 ± 0.51	0.93 ± 0.04	0.89 ± 0.06	12.19 ± 0.45	0.28
P1	Xylose	45.93 ± 0.64	0.35	12.36 ± 0.36	0.67 ± 0.02	1.29 ± 0.08	12.75 ± 0.41	0.27
	Glucose	48.36 ± 0.51	0.63	12.87 ± 0.27	1.05 ± 0.03	0.86 ± 0.04	12.59 ± 0.36	0.27
	Sucrose	47.63 ± 0.32	0.57	13.49 ± 0.34	1.24 ± 0.05	0.98 ± 0.02	13.18 ± 0.38	0.28
P2	Xylose	35.57 ± 0.52	0.23	8.75 ± 0.37	1.16 ± 0.04	0.82 ± 0.03	12.54 ± 0.27	0.25
	Glucose	46.55 ± 0.42	0.60	12.38 ± 0.26	0.83 ± 0.02	0.89 ± 0.04	11.76 ± 0.42	0.27
	Sucrose	47.45 ± 0.31	0.55	13.12 ± 0.32	0.96 ± 0.04	0.91 ± 0.05	12.57 ± 0.32	0.28
P3	Xylose	34.85 ± 0.61	0.26	7.18 ± 0.29	0.63 ± 0.06	0.94 ± 0.03	13.02 ± 0.37	0.21
	Glucose	44.82 ± 0.28	0.56	12.40 ± 0.38	0.93 ± 0.02	0.98 ± 0.04	12.70 ± 0.36	0.28
	Sucrose	47.16 ± 0.33	0.57	13.29 ± 0.17	0.81 ± 0.03	0.76 ± 0.05	12.79 ± 0.33	0.28
P4	Xylose	48.73 ± 0.49	0.48	13.72 ± 0.32	0.75 ± 0.02	1.52 ± 0.04	13.53 ± 0.42	0.28
	Glucose	48.13 ± 0.32	0.64	12.78 ± 0.29	0.88 ± 0.03	1.04 ± 0.07	12.48 ± 0.39	0.27
	Sucrose	47.79 ± 0.55	0.59	13.63 ± 0.59	0.79 ± 0.03	0.93 ± 0.05	13.34 ± 0.27	0.29

Data are given as mean ± standard error value of each group (*p* < 0.05)

Table 3 shows that melanin synthesis can be stimulated by utilizing xylose as carbon source, whereas a smaller amount of melanin was produced when sucrose was the carbon source. The maximum carbon source consumption rate of all strains showed no marked difference when glucose and sucrose were utilized as carbon source. P4 yielded the highest maximum xylose consumption rate (0.48 g/h), whereas the lowest xylose consumption rate was achieved by P2 (0.23 g/h). Furthermore, the heavy oil production of the mutants was not affected by the introduction of the xylose utilization pathway.

### Fermentation performance of the P4 strain on mimicked hydrolysate

**Fig. 5:**
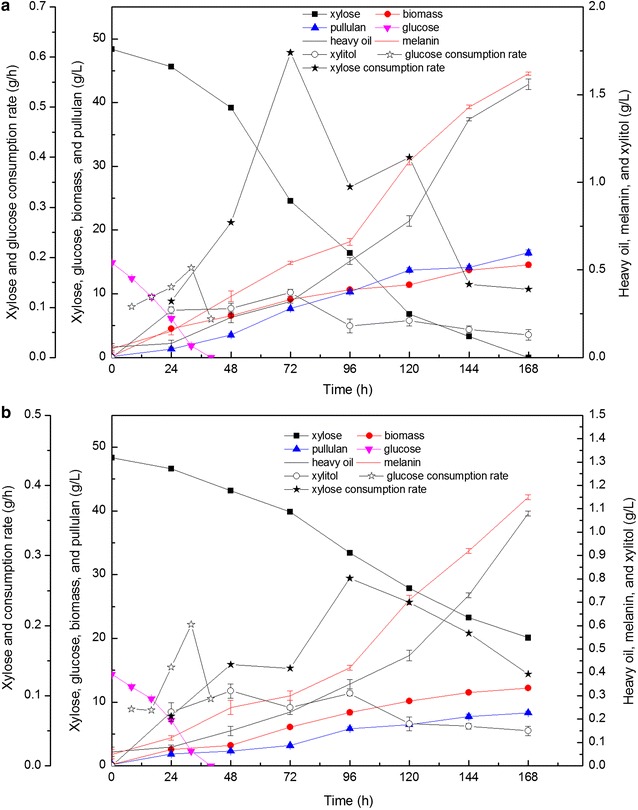
Fermentation performances of P4 mutant (**a**) and parent strain (**b**) utilizing mimicked hydrolysate as the carbon source. Data are given as mean ± standard error value of each group (*p* < 0.05)

The strain of P4 consumed the highest amount of xylose relative to other mutant strains. For further understanding its characteristics, the fermentation performances of P4 were fully compared with the parent strain utilizing mimicked hydrolysate as carbon source (Fig. 5). In the presence of glucose, the xylose-consuming capacity of the parent strain was markedly inhibited (Fig. 5a), whereas the P4 mutant was not affected. Both strains can utilize glucose efficiently, and glucose was consumed within 40 h. After 168 h of fermentation, the residue xylose in the fermentation broth of the parent strain and P4 mutant were 20.14 and 0 g/L, respectively. The maximum xylose consumption rate of P4 reached 0.61 g/h, whereas only 0.27 g/h was achieved by the parent strain (Fig. 5). This result demonstrated the superior xylose consumption capability of P4 in comparison with the parent strain. The xylitol production of P4 was identical to that of the parent strain (Fig. 5). Furthermore, the production of pullulan, heavy oil, and melanin of the P4 mutant increased by 97.72, 44.44, and 47%, respectively, compared with that of the parent strain.

This study represents the first attempt to enhance xylose-consuming capacity through genetic approach. Our previous study [14] aimed to improve erythritol production from xylose by *A. pullulans*. Random mutagenesis was applied, resulting in a 21% increase in xylose consumption. In this study, 17.76% increase in consumed xylose by P4 was obtained through metabolic engineering. Traditional microbe breeding requires laborious work and may lead to uncertain changes in genotype and phenotype [31]. By contrast, metabolic engineering can overcome these drawbacks. The results obtained in this study may provide guidance for the biotransformation of xylose-rich raw materials to high-value-added products through the genetic manipulation of *A. pullulans*.

## Conclusion

*Spathaspora passalidarum* XR–XDH and *Piromyces* sp. XI pathways were constructed and compared in *A. pullulans*. The results indicated that the P4 mutant with overexpressed *XYL1.2* and *XYL2.2* consumed the largest amount of xylose, whereas the introduction of the *Piromyces* sp. XI pathway in *A. pullulans* failed to enhance the xylose fermentation capacity. Increased pullulan production and intracellular reducing equivalents were observed in mutants with improved xylose-consuming capacity. The mutant with overexpressed *XYL1.2* and *XYL2.2* exhibited good fermentation performance with mimicked hydrolysate. The present study may be used as a reference to improve the xylose-consuming capabilities of other chassis organisms.

## Additional files

**Additional file 1: Table S1.** Primers used for genetic manipulation of *A. pullulans* var. *melanogenum* CBS 110374.

**Additional file 2: Figure S1.** The sketch map for knock-in of XI gene by one-step homologous recombination-based method. **Figure S2.** PCR verification of the transformant with overexpressed XI gene. **Figure S3.** PCR verification of the transformant with overexpressed XYL1.1 (A), XYL1.2 (B), XYL2.1 (C) and XYL2.2 (D). **Figure S4.** PCR verification of the transformant with overexpressed XYL1.1 and XYL2.1 (A), XYL1.1 and XYL2.2 (B), XYL1.2 and XYL2.1 (C), XYL1.2 and XYL2.2 (D). **Figure S5.** Comparing the production of pullulan, heavy oil and melanin of the strains with single overexpressed XR gene or XDH gene with that of the parent strain.

## References

[1] Chi Z, Wang F, Chi Z, Yue L, Liu G, Zhang T (2009). Bioproducts from *Aureobasidium pullulans*, a biotechnologically important yeast. Appl Microbiol Biotechnol.

[2] Liu YY, Chi Z, Wang ZP, Liu GL, Chi ZM (2014). Heavy oils, principally long-chain *n*-alkanes secreted by *Aureobasidium pullulans* var. *melanogenum* strain P5 isolated from mangrove system. J Ind Microbiol Biotechnol.

[3] Wang WL, Chi ZM, Chi Z, Li J, Wang XH (2009). Siderophore production by the marine-derived *Aureobasidium pullulans* and its antimicrobial activity. Bioresour Technol.

[4] FitzPatrick M, Champagne P, Cunningham MF, Whitney RA (2010). A biorefinery processing perspective: treatment of lignocellulosic materials for the production of value-added products. Bioresour Technol.

[5] Karhumaa K, Fromanger R, Hahn-Hägerdal B, Gorwa-Grauslund MF (2007). High activity of xylose reductase and xylitol dehydrogenase improves xylose fermentation by recombinant *Saccharomyces cerevisiae*. Appl Microbiol Biotechnol.

[6] Lee SM, Jellison T, Alper HS (2014). Systematic and evolutionary engineering of a xylose isomerase-based pathway in *Saccharomyces cerevisiae* for efficient conversion yields. Biotechnol Biofuels.

[7] Weyda I, Lübeck M, Ahring BK, Lübeck PS (2014). Point mutation of the xylose reductase (XR) gene reduces xylitol accumulation and increases citric acid production in *Aspergillus carbonarius*. J Ind Microbiol Biotechnol.

[8] Hou J, Vemuri GN, Bao X, Olsson L (2009). Impact of overexpressing NADH kinase on glucose and xylose metabolism in recombinant xylose-utilizing *Saccharomyces cerevisiae*. Appl Microbiol Biotechnol.

[9] Nguyen NH, Suh SO, Marshall CJ, Blackwell M (2006). Morphological and ecological similarities: wood-boring beetles associated with novel xylose-fermenting yeasts, *Spathaspora passalidarum* gen. sp. nov. and *Candida jeffriesii* sp. nov. Mycol Res.

[10] Yu H, Guo J, Chen Y, Fu G, Li B, Guo X, Xiao D (2017). Efficient utilization of hemicellulose and cellulose in alkali liquor-pretreated corncob for bioethanol production at high solid loading by *Spathaspora passalidarum* U1-58. Bioresour Technol.

[11] Teunissen AWRH, Bont JAMD. Xylose isomerase genes and their use in fermentation of pentose sugars. 2010, WO Patent WO 2010/074577.

[12] Maris A, Winkler A, Kuyper M, de Laat W, van Dijken J, Pronk J (2007). Development of efficient xylose fermentation in *Saccharomyces cerevisiae*: xylose isomerase as a key component. Biofuels..

[13] Gostinčar C, Ohm RA, Kogej T, Sonjak S, Turk M, Zajc J, Sharma A (2014). Genome sequencing of four *Aureobasidium pullulans* varieties: biotechnological potential, stress tolerance, and description of new species. BMC Genom.

[14] Guo J, Wang Y, Li B, Huang S, Chen Y, Guo X, Xiao D (2017). Development of a one-step gene knock-out and knock-in method for metabolic engineering of *Aureobasidium pullulans*. J Biotechnol.

[15] Dmytruk OV, Voronovsky AY, Abbas CA, Dmytruk KV, Ishchuk OP, Sibirny AA (2007). Overexpression of bacterial xylose isomerase and yeast host xylulokinase improves xylose alcoholic fermentation in the thermotolerant yeast *Hansenula polymorpha*. FEMS Yeast Res.

[16] Bengtsson O, Hahn-Hägerdal B, Gorwa-Grauslund MF (2009). Xylose reductase from *Pichia stipitis* with altered coenzyme preference improves ethanolic xylose fermentation by recombinant *Saccharomyces cerevisiae*. Biotechnol Biofuels.

[17] Chen Y, Guo J, Li F, Liu M, Zhang X, Guo X, Xiao D (2014). Production of pullulan from xylose and hemicellulose hydrolysate by *Aureobasidium pullulans* AY82 with pH control and dl-dithiothreitol addition. Biotechnol Bioprocess Eng.

[18] Zheng W, Campbell BS, McDougall BM, Seviour RJ (2008). Effects of melanin on the accumulation of exopolysaccharides by *Aureobasidium pullulans* grown on nitrate. Bioresour Technol.

[19] Guo X, Cao C, Wang Y, Li C, Wu M, Chen Y, Xiao D (2014). Effect of the inactivation of lactate dehydrogenase, ethanol dehydrogenase, and phosphotransacetylase on 2,3-butanediol production in *Klebsiella pneumoniae* strain. Biotechnol Biofuels.

[20] Zhang B, Zhang J, Wang D, Han R, Ding R, Gao X, Hong J (2016). Simultaneous fermentation of glucose and xylose at elevated temperatures co-produces ethanol and xylitol through overexpression of a xylose-specific transporter in engineered *Kluyveromyces marxianus*. Bioresour Technol.

[21] Zou X, Yang J, Tian X, Guo M, Li Z, Li Y (2016). Production of polymalic acid and malic acid from xylose and corncob hydrolysate by a novel *Aureobasidium pullulans* YJ 6–11 strain. Process Biochem.

[22] Kuyper M, Harhangi HR, Stave AK, Winkler AA, Jetten MS, de Laat WT, Pronk JT (2003). High-level functional expression of a fungal xylose isomerase: the key to efficient ethanolic fermentation of xylose by *Saccharomyces cerevisiae*?. FEMS Yeast Res.

[23] Kuyper M, Winkler AA, van Dijken JP, Pronk JT (2004). Minimal metabolic engineering of *Saccharomyces cerevisiae* for efficient anaerobic xylose fermentation: a proof of principle. FEMS Yeast Res.

[24] Zhou H, Cheng JS, Wang BL, Fink GR, Stephanopoulos G (2012). Xylose isomerase overexpression along with engineering of the pentose phosphate pathway and evolutionary engineering enable rapid xylose utilization and ethanol production by *Saccharomyces cerevisiae*. Metab Eng.

[25] Khoury GA, Fazelinia H, Chin JW, Pantazes RJ, Cirino PC, Maranas CD (2009). Computational design of *Candida boidinii* xylose reductase for altered cofactor specificity. Protein Sci.

[26] Hou X (2012). Anaerobic xylose fermentation by *Spathaspora passalidarum*. Appl Microbiol Biotechnol.

[27] Cadete RM, Alejandro M, Sandström AG, Ferreira C, Gírio F, Gorwa-Grauslund MF, Fonseca C (2016). Exploring xylose metabolism in *Spathaspora* species: *XYL1.2* from *Spathaspora passalidarum* as the key for efficient anaerobic xylose fermentation in metabolic engineered *Saccharomyces cerevisiae*. Biotechnol Biofuels.

[28] Su YK, Willis LB, Jeffries TW (2015). Effects of aeration on growth, ethanol and polyol accumulation by *Spathaspora passalidarum* NRRL Y-27907 and *Scheffersomyces stipitis* NRRL Y-7124. Biotechnol Bioeng.

[29] Han S, Gao XY, Ying HJ, Zhou CC (2016). NADH gene manipulation for advancing bioelectricity in *Clostridium ljungdahlii* microbial fuel cells. Green Chem.

[30] Sheng L, Tong Q, Ma M (2016). Why sucrose is the most suitable substrate for pullulan fermentation by *Aureobasidium pullulans* CGMCC1234?. Enzyme Microb Technol.

[31] Chen X, Gao C, Guo L, Hu G, Luo Q, Liu J, Liu L (2017). DCEO biotechnology: tools to design, construct, evaluate, and optimize the metabolic pathway for biosynthesis of chemicals. Chem Rev.

