# Supplementary material for: Heterologous expression of Spathaspora passalidarum xylose reductase and xylitol dehydrogenase genes improved xylose fermentation ability of Aureobasidium pullulans
Source: Microb Cell Fact. 2018 Apr 30;17:64. doi: 10.1186/s12934-018-0911-1 (PMC5925849; doi:10.1186/s12934-018-0911-1)
Supplement: Supplementary file 1 — Additional file 1: Table S1. Primers used for genetic manipulation of A. pullulans var. melanogenum CBS 110374. [file 12934_2018_911_MOESM1_ESM.docx]

Table S1 Primers used for genetic manipulation of *A. pullulans* var. *melanogenum* CBS 110374

| Primer name | | Primer sequence (5′→3′) | | |
| --- | --- | --- | --- | --- |
| 28U1 | | AAGAGTAAGGGTGCTCAGCG | | |
| 28U2 | | GACAGGCTGGATCACTTTCCCTTCTATGCCCCTCGGAATACCAAG | | |
| HPTF | GCTGACAGCATACCAACATCTCTTGTCAAGACAGCAAGAACGGG | | |  |
| HPTR | GCCCGTAAATGTATCTCGAGTTCAATCTTCCCTTTCACTAGGTCG | | |  |
| PGKpF | CTGTCCGACCTAGTGAAAGGGAAGACATTGCCTCACTTAGGTAACC | | |  |
| PGKpR | | TTTGTGGGAAATATTCCTTAGCCATTGTGACTGAATCGAGTGTGTCAG | | |
| PGKp1R | | CTGAGGATAATTTAATAGTAGCCATTGTGACTGAATCGAGTGTGTCAG | | |
| PGKp2R | | AACCTGAAGATAATTTAAAAGACATTGTGACTGAATCGAGTGTGTCAG | | |
| XI1 | | GTCTGACACACTCGATTCAGTCACAATGGCTAAGGAATATTTCCCACA | | |
| XI2 | | TTTTGAAAAAAACACTCTGATGAGTTTATTGGTACATGGCAAC | | |
| XR1U | | GTCTGACACACTCGATTCAGTCACAATGGCTACTATTAAATTATC | | |
| XR1D | | TTTTGAAAAAAACACTCTGATGAGTATAGTTAACTATGTCACTTG | | |
| XR2U | | GTCTGACACACTCGATTCAGTCACAATGTCTTTTAAATTATCTTCAGG | | |
| XR2D | | TTTTGAAAAAAACACTCTGATGAGTTTAAACAAAGATTGGAATATGGTC | | |
| PGKtF | | TTATTGGTACATGGCAACAATAGCTACTCATCAGAGTGTTTTTTTC | | |
| PGKt1F | | ATAGTTAACTATGTCACTTGAACTCACTCATCAGAGTGTTTTTTTC | | |
| PGKt2F | | TTAAACAAAGATTGGAATATGGTCCACTCATCAGAGTGTTTTTTTC | | |
| PGKt1R | | GACGGCACCCAATACCAAGCATAGCCTCAAAAATGGTTAAAAGAC | | |
| PGKtR | | TCGCAGTTGGTATATGGGCAGTTTCCTCAAAAATGGTTAAAAGAC | | |
| GAPps | | CTCAAAAATGGTTAAAAGACTGATCGAAACTGCCCATATACCAAC | | |
| GAPp1a | | GAACTAATGATGGGTTAGCAACCATGTTTATGTGTCTGCGGGGTAG | | |
| GAPp2a | | GCACTAAAGAGGGATTAGCAACCATGTTTATGTGTCTGCGGGGTAG | | |
| XDH1U | | GTTTATGTGTCTGCGGGGTAGATGTATGGTTGCTAACCCATCATTAG | | |
| XDH1D | | GCTTGGTCATACGCACATCAGAGATTTATAATGGACCATCAATCAAAC | | |
| XDH2U | | GTTTATGTGTCTGCGGGGTAGATGTATGGTTGCTAATCCCTCTTTA | | |
| XDH2D | | GCTTGGTCATACGCACATCAGAGATCTACTCTGGTCCATCAATTAAAC | | |
| GAPt1s | | TTATAATGGACCATCAATCAAACACATCTCTGATGTGCGTATGAC | | |
| GAPt2s | | CTACTCTGGTCCATCAATTAAACACATCTCTGATGTGCGTATGAC | | |
| GAPta | | GACGGCACCCAATACCAAGCATAGCCTAAGGTCATGGTTTCTCTG | | |
| 28D1 | | CTCAAAAATGGTTAAAAGACTGATCGCTATGCTTGGTATTGGGTG | | |
| 28D2 | | CTAAGGTCATGGTTTCTCTGGGCAGGCTATGCTTGGTATTGGGTG | | |
| 28D3 | | CTATGACGTCCTGTTCCAAG | | |
| PGKt3R | | GACGGCACCCAATACCAAGCATAGCCTCAAAAATGGTTAAAAGAC | | |
| 28D4 | | CTCAAAAATGGTTAAAAGACTGATCGCTATGCTTGGTATTGGGTG | | |
| GAPp3s | | CTGTCCGACCTAGTGAAAGGGAAGAGAAACTGCCCATATACCAAC | | |
| HPT1R | | TCGCAGTTGGTATATGGGCAGTTTCTCTTCCCTTTCACTAGGTCG | | |
| yXR1a | CTCTTTAAACCGTCACCGACTTC | |  |  |
| yXR1s | GCAATGGTAGCAATATTAATTG | |  |  |
| yXR2a | CATCGAATGATCTGATACCAC | |  |  |
| yXR2s | GATACTCCAGAAAGATTGGTC | |  |  |
| yXDH1a | CTTCGACAATAACGTCGGTTG | |  |  |
| yXDH2a | CTTGGACAATCACATCGGTG | |  |  |

For *in vivo* homologous recombination-based method, all the overlaps (underlined bases) were shown as 50 bp.
