# Supplementary material for: Heterologous expression of Spathaspora passalidarum xylose reductase and xylitol dehydrogenase genes improved xylose fermentation ability of Aureobasidium pullulans
Source: Microb Cell Fact. 2018 Apr 30;17:64. doi: 10.1186/s12934-018-0911-1 (PMC5925849; doi:10.1186/s12934-018-0911-1)
Supplement: Supplementary file 2 — Additional file 2: Figure S1. The sketch map for knock-in of XI gene by one-step homologous recombination-based method. Figure S2. PCR verification of the transformant with overexpressed XI gene. Figure S3. PCR verification of the transformant with overexpressed XYL1.1 (A), XYL1.2 (B), XYL2.1 (C) and XYL2.2 (D). Figure S4. PCR verification of the transformant with overexpressed XYL1.1 and XYL2.1 (A), XYL1.1 and XYL2.2 (B), XYL1.2 and XYL2.1 (C), XYL1.2 and XYL2.2 (D). Figure S5. Comparing the production of pullulan, heavy oil and melanin of the strains with single overexpressed XR gene or XDH gene with that of the parent strain. [file 12934_2018_911_MOESM2_ESM.docx]

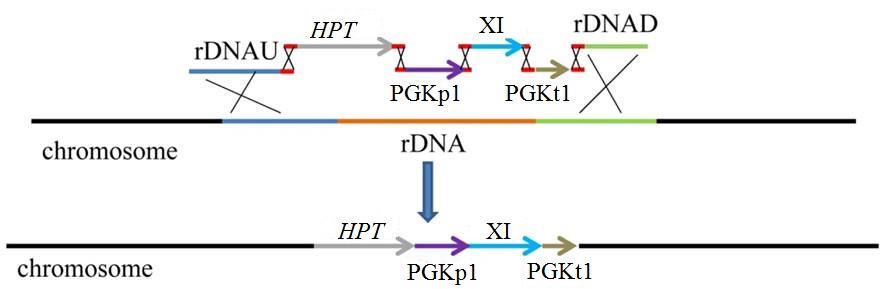
 **Figure S1** The sketch map for knock-in of XI gene by one-step homologous recombination-based method


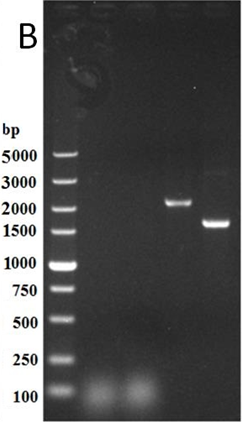


**Figure S2** PCR verification of the transformant with overexpressed *XI*

M, DL5000 marker; 1, Result from CBS 110374 amplified with 28U1/H1 primers; 2, Result from CBS 110374 amplified with H2/28D3 primers; 3, Result from X5 amplified with 28U1/H1 primers (2245 bp); 4, Result from X5 amplified with H2/28D3 primers (1783 bp)


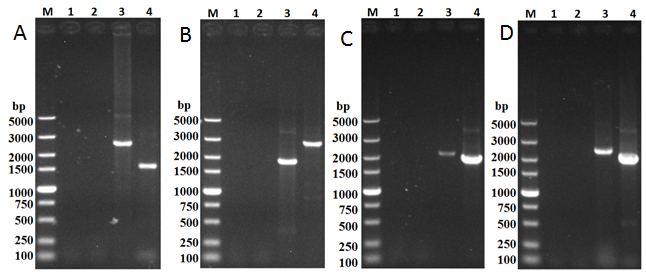


**Figure S3** PCR verification of the transformant with overexpressed *XYL1.1* (A), *XYL1.2* (B), *XYL2.1* (C) and *XYL2.2* (D)

A: M, DL5000 marker; 1, Result from CBS 110374 amplified with 28U1/yXR1a primers; 2, Result from CBS 110374 amplified with XR1s/28D3 primers; 3, Result from the transformant with overexpressed *XYL1.1* amplified with 28U1/yXR1 primers (2675 bp); 4, Result from the transformant with overexpressed *XYL1.1* amplified with XR1s/28D3 primers (1579 bp)

B: M, DL5000 marker; 1, Result from CBS 110374 amplified with 28U1/yXR1a primers; 2, Result from CBS 110374 amplified with XR1s/28D3 primers; 3, Result from the transformant with overexpressed *XYL1.2* amplified with 28U1/yXR1 primers (1836 bp); 4, Result from the transformant with overexpressed *XYL1.2* amplified with XR1s/28D3 primers (2763 bp)

C: M, DL5000 marker; 1, Result from CBS 110374 amplified with 28U1/yXDH1a primers; 2, Result from CBS 110374 amplified with XDH1s/28D3 primers; 3, Result from the transformant with overexpressed *XYL2.1* amplified with 28U1/yXDH1a primers (2259 bp); 4, Result from the transformant with overexpressed *XYL2.1* amplified with XDH1s/28D3 primers (1858 bp)

D: M, DL5000 marker; 1, Result from CBS 110374 amplified with 28U1/yXDH2a primers; 2, Result from CBS 110374 amplified with XDH2s/28D3 primers; 3, Result from the transformant with overexpressed *XYL2.2* amplified with 28U1/yXDH2a primers (2416 bp); 4, Result from the transformant with overexpressed *XYL2.2* amplified with XDH2s/28D3 primers (1774 bp)


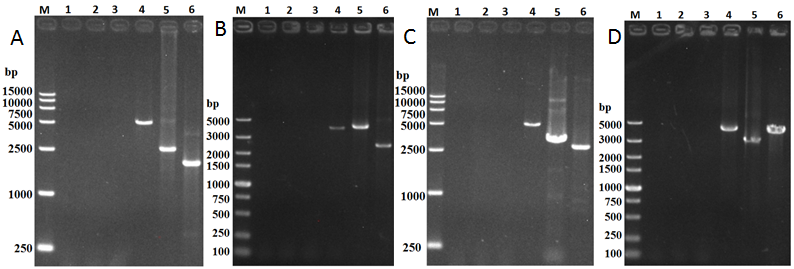


**Figure S4** PCR verification of the transformant with overexpressed *XYL1.1* and *XYL2.1* (A), *XYL1.1* and *XYL2.2* (B), *XYL1.2* and *XYL2.1* (C), *XYL1.2* and *XYL2.2* (D)

A: M, DL5000 marker; 1, Result from CBS 110374 amplified with 28U1/yXR1a primers; 2, Result from CBS 110374 amplified with yXR1s/yXDH1a primers; 3, Result from CBS 110374 amplified with XDH1U/28D3 primers; 4, Result from the transformant with overexpressed *XYL1.1* and *XYL2.1* amplified with 28U1/yXR1a primers (5328 bp); 5, Result from the transformant with overexpressed *XYL1.1* and *XYL2.1* amplified with yXR1s/yXDH1a primers (2425 bp); 6, Result from the transformant with overexpressed *XYL1.1* and *XYL2.1* amplified with XDH1U/28D3 primers (1852 bp)

B: M, DL5000 marker; 1, Result from CBS 110374 amplified with 28U1/yXR1a primers; 2, Result from CBS 110374 amplified with yXR1s/yXDH2a primers; 3, Result from CBS 110374 amplified with XDH2U/28D3 primers; 4, Result from the transformant with overexpressed *XYL1.1* and *XYL2.2* amplified with 28U1/yXR1a primers (3435 bp); 5, Result from the transformant with overexpressed *XYL1.1* and *XYL2.2* amplified with yXR1s/yXDH2a primers (3518 bp); 6, Result from the transformant with overexpressed *XYL1.1* and *XYL2.2* amplified with XDH2U/28D3 primers (2143 bp)

C: M, DL5000 marker; 1, Result from CBS 110374 amplified with 28U1/yXR2a primers; 2, Result from CBS 110374 amplified with yXR2s/yXDH1a primers; 3, Result from CBS 110374 amplified with XDH1U/28D3 primers; 4, Result from the transformant with overexpressed *XYL1.2* and *XYL2.1* amplified with 28U1/yXR2a primers (4819 bp); 5, Result from the transformant with overexpressed *XYL1.2* and *XYL2.1* amplified with yXR2s/yXDH1a primers (3741 bp); 6, Result from the transformant with overexpressed *XYL1.2* and *XYL2.1* amplified with XDH1U/28D3 primers (2635 bp)

D: M, DL5000 marker; 1, Result from CBS 110374 amplified with 28U1/yXR2a primers; 2, Result from CBS 110374 amplified with yXR2s/yXDH2a primers; 3, Result from CBS 110374 amplified with XDH2U/28D3 primers; 4, Result from the transformant with overexpressed *XYL1.2* and *XYL2.2* amplified with 28U1/yXR2a primers (3625 bp); 5, Result from the transformant with overexpressed *XYL1.2* and *XYL2.2* amplified with yXR2s/yXDH2a primers (2673 bp); 6, Result from the transformant with overexpressed *XYL1.2* and *XYL2.2* amplified with XDH2U/28D3 primers (3518 bp)


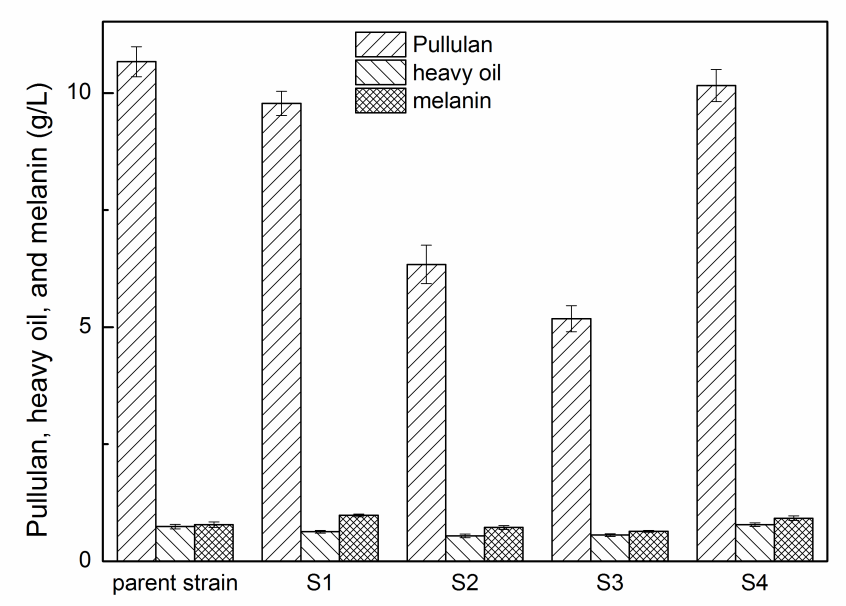


**Figure S5** Comparing the production of pullulan, heavy oil and melanin of the strains with single overexpressed XR gene or XDH gene with that of the parent strain. Data are given as mean ± standard error value of each group (*p* < 0.05)
